# Application of Protein Structure Encodings and Sequence Embeddings for Transporter Substrate Prediction

**DOI:** 10.3390/molecules30153226

**Published:** 2025-08-01

**Authors:** Andreas Denger, Volkhard Helms

**Affiliations:** Center for Bioinformatics, Saarland University, 66123 Saarbrücken, Germany

**Keywords:** membrane transport, membrane bioinformatics, substrate prediction, protein function prediction, deep learning, machine learning, AlphaFold, protein language model, gene ontology, feature extraction

## Abstract

Membrane transporters play a crucial role in any cell. Identifying the substrates they translocate across membranes is important for many fields of research, such as metabolomics, pharmacology, and biotechnology. In this study, we leverage recent advances in deep learning, such as amino acid sequence embeddings with protein language models (pLMs), highly accurate 3D structure predictions with AlphaFold 2, and structure-encoding 3Di sequences from FoldSeek, for predicting substrates of membrane transporters. We test new deep learning features derived from both sequence and structure, and compare them to the previously best-performing protein encodings, which were made up of amino acid k-mer frequencies and evolutionary information from PSSMs. Furthermore, we compare the performance of these features either using a previously developed SVM model, or with a regularized feedforward neural network (FNN). When evaluating these models on sugar and amino acid carriers in *A. thaliana*, as well as on three types of ion channels in human, we found that both the DL-based features and the FNN model led to a better and more consistent classification performance compared to previous methods. Direct encodings of 3D structures with Foldseek, as well as structural embeddings with ProstT5, matched the performance of state-of-the-art amino acid sequence embeddings calculated with the ProtT5-XL model when used as input for the FNN classifier.

## 1. Introduction

Due to advances in high-throughput sequencing and proteomics, the amount of biological data is growing exponentially. This enables a better understanding of biological complexity by revealing new protein families with common evolutionary origins, as well as shared three-dimensional folds derived from predicted protein structures. At the same time, it also enhances the possibilities for encoding this data, in order to sharpen the accuracy of predictive machine learning models. These models can, in turn, help us understand the biological functions that these genes and gene products serve in the living organism.

### 1.1. Motivation and Background

Membrane transporters are a crucial part of any cell, selectively transporting nutrients, metabolites, signaling molecules, and charged ions across its membranes, as well as drugs and xenobiotics. Determining the transport proteins that are present in a proteome, along with their transport mechanisms and their transported substrates, is therefore an important task that leads towards a deeper understanding of the connections between intra- and inter-cellular networks. Discovery of transporters for a particular substrate has implications for various fields, such as pharmacology and metabolomics. In biotechnology, the efflux of intermediary molecules is a common problem when optimizing micro-organisms for the production of a particular product.

After analyzing predicted 3D structures of human solute carriers, along with their homologues in other species, Ferrada and Superti-Furga [1] suggested that the 455 proteins share a total of 180 evolutionary origins. Furthermore, most of these proteins can be assigned to one of a small number of different structural folds [2,3]. The substrate specificity, however, does not necessarily depend on the fold and evolutionary origin of the protein, as it can evolve later throughout evolution. Human sugar transporters, for example, include the GLUT family of uniporters [4], which possess the MFS fold [5], while the SGLT family of sugar-cation symporters displays the LeuT fold, making it part of the APC superfamily, which is mainly made up of amino acid transporter families [6]. In 2010 [7], the SWEET family of bi-directional sugar uniporters was found in *A. thaliana*, and homologues have been discovered in prokaryotes and animals, including one human SWEET transporter [8]. Prokaryotes also possess ABC transport complexes with unique folds that import nutrients, such as amino acids and sugars [9].

### 1.2. Membrane Transporter Substrate Prediction with Machine Learning

Due to this complexity, one cannot solely rely on similarity of sequence and structure to tell transport proteins apart by their substrates, and there is a need for developing specialized protein encodings that can capture the necessary information, beyond evolutionary relationships and shared protein domains. Machine learning methods trained on protein sequence have successfully been used for this purpose. Schaadt and Helms [10] encoded protein sequences through amino acid frequencies, as well as through data from multiple sequence alignments (MSAs) [11], to assign transporters in *A. thaliana* to four substrate classes. Other approaches incorporated evolutionary information from position-specific scoring matrices (PSSMs) [12], as well as physicochemical properties of residues in the sequence [13,14], to classify transporters in manually assembled subsets of Swiss-Prot. More recently, deep learning architectures have also been applied in this field. For example, the tool SPOT used transformer networks to predict whether an arbitrary chemical entity is likely to be a substrate of a given transporter or not [15]. The tool NA_cNN tested four current protein language model embeddings of amino acid sequences, and was able to identify sodium transporters among all membrane proteins with high accuracy [16].

In a previous study [17], we compared and combined existing protein sequence features into a META-feature, and tested their performance across different machine learning models and transporter datasets. We then compared model performance to various similarity measures between substrate classes and protein annotations through correlation analysis and statistical testing, and introduced an optimization algorithm that identifies a set of substrate classes for a dataset of transmembrane transporters with minimal redundancy and high pairwise classification performance [18]. As a basis for that work, we also developed a dataset pipeline for creating annotated protein sets, where constraints on predicted and experimental data allowed for a trade-off between the quantity and quality of the training data.

### 1.3. Applications of Deep Learning Models in Bioinformatics

Recent advances in deep learning, such as transformer models [19], convolutional neural networks (CNNs) [20], graph neural networks (GNNs) [21], and variational autoencoders (VAEs) [22], have successfully been applied to a wide range of tasks in computational biology. This includes feature extraction from protein sequences [23,24], protein sequence generation from text [25,26,27], as well as various predictive tasks, e.g., for secondary and tertiary structure [28,29,30], structural similarity and protein family membership [31,32], GO term annotations [33,34,35,36,37], protein–protein interactions (PPIs) [38,39], sequence variant effects [40,41], sub-cellular location [42,43], and many more.

Transformer-based protein language models (pLMs), such as ProtT5 [23] and ESM [24], have been trained on proteomics datasets containing a very large number of sequences [37]. These pre-trained models can now be used for feature extraction from amino acid sequences in the form of language embeddings, or they can be fine-tuned, e.g., for protein sequence generation [44]. To apply these methods from natural language processing (NLP) to proteins, each amino acid in the sequence is treated as a word, and encoded by a numerical token. The encoder of the transformer model then converts these words into fixed-length numerical vectors. These vectors can be averaged (mean-pooled) across the sequence to create a vector representation for the entire protein. Both the per-protein and per-residue embeddings are suitable as input for other models, to predict properties of the entire protein or of individual sequence positions, respectively, through supervised learning [35,36,37,43,44]. As similar proteins produce similar embeddings, they can also be used for unsupervised learning techniques such as clustering, and for visualization [45]. In addition to embeddings, another commonly found method for encoding protein sequences is that of trigrams [46,47], which we refer to as 3-mer frequencies. Previous approaches for membrane transporter substrate prediction trained models on *k*-mer frequencies with k∈{1,2} in the form of amino acid composition and pair amino acid composition (AAC and PAAC, respectively). Yet, it would be interesting to compare the performance of k=3.

### 1.4. Predictive Methods Based on Protein Structures

Due to the high accuracy of protein 3D structural models predicted from sequences with AlphaFold [28] and similar models [29,30], and the resulting availability of protein structures for hundreds of millions of proteins [48], a new generation of bioinformatics software has emerged that uses this data for data mining and prediction, often along with features derived from the amino acid sequence, from evolutionary information in the form of PSSMs or MSAs, and/or from known protein–protein interactions (PPIs) [49,50,51,52]. Some methods, such as DeepFRI [50], GAT-GO [52], GNNGO3D [49], and Panda3D [51], encode the structures directly, typically by calculating contact maps or distance maps between α-carbon atoms in the amino acids. Another technique for encoding protein structures, which is already used by some homology-based predictive models [53,54], exists in the form of 3Di sequences.

These 3Di sequences were introduced together with the 3D structure search tool Foldseek [54], and aim to encode a protein structure as a structural sequence that has the same length as the respective amino acid sequence, and the same alphabet size of 20. The 3Di state of a residue takes its nearest neighbor into account, along with surrounding positions in the sequence. Based on descriptors derived from these residues and their relative positions in the structure and sequence, a VAE model finds 20 discrete states with high conservation among the protein structures in its training dataset. The 3Di sequence of a protein can then be used for sequence alignments with the help of a substitution matrix to compare the folds of two proteins, or for fast database searches to find similar structures, and potentially infer functions or properties through structural homology [53,54].

So far, 3Di sequences have only been used as ML training data by a few predictive models, e.g., the Topsy-Turvy 3D (TT3D) model [55] for predicting PPIs, which encoded 3Di sequences by one-hot encoding, and combined that with amino acid embeddings from a pre-trained pLM. Furthermore, the ProstT5 model [56] is a Text-to-Text Transfer Transformer (T5) [57] that aims to translate directly between amino acid and 3Di sequences, and was trained on both. As it is a transformer model, its encoder can be used to create embeddings for either type of sequence.

### 1.5. Outline

The goal of this study is to test whether recent advances in deep learning lead to better models for membrane transporter substrate prediction. To this end, we compared previously published methods, which train SVMs on amino acid k-mer frequencies and evolutionary information from PSSMs, against features generated with various deep learning models, such as amino acid embeddings from pLMs, as well as features derived from 3Di sequences. The structural models for calculating the 3Di sequences were, in turn, predicted from the protein sequence with AlphaFold. We tested the performance of 3Di embeddings with ProstT5, and used 3Di k-mer frequencies as ML-features for prediction, both of which, to our knowledge, have not been tested so far. Finally, we tested the impact of replacing our previous SVM model with a regularized feedforward neural network (FNN).

## 2. Results

### 2.1. Classifying Substrates of Solute Carriers in A. thaliana

#### 2.1.1. Dataset Analysis

As a first task for the new machine learning features, we trained a model to distinguish between amino acid transporters and sugar transporters in *A. thaliana*. We used our dataset creation pipeline (see Section 3.2 and Section 3.3) to generate a dataset of 33 amino acid and 32 sugar transporters, excluding those where protein data and GO annotations were not manually curated. After applying sequence clustering at a 70% threshold, the numbers of sugar and amino acid transporters were reduced to 28 and 26, respectively. The raw data for all ML-features was available for every transporter.

According to InterPro [58], 13 of these sugar transporters were members of the SWEET (Sugars Will Eventually be Exported) family (TCDB accession 2.A.123), 15 were part of the Major Facilitator Superfamily (MFS, TCDB 2.A.1), and one protein (SWEET4) was annotated with both. MFS and SWEET proteins are structurally distinct, and contain a different number of transmembrane helices (TMHs). SWEET4 contains seven TMHs arranged into two bundles of three TMHs with one connecting helix in the middle, as is typical for members of the SWEET family, whereas the MFS superfamily is defined by its characteristic twelve TMHs arranged into two bundles of six [5,8]. The nucleotide sugar transporter URGT6 was the only protein in the dataset that was annotated with neither family. When looking at the names of the MFS proteins, we found that most transport hexoses and pentoses, most commonly as uniporters, but two xylose-proton symporters exist as well. In addition to the aforementioned substrates, there was one transporter each for sucrose, polyol, and ascorbate, which are grouped under sugar transmembrane transport by the Gene Ontology.

The amino acid transporters were not as thoroughly annotated with family names. The *amino acid transporter, transmembrane domain* (InterPro accession IPR013057) was found in 13 out of 26 proteins. The remainder included four members of the mitochondrial carrier domain superfamily, and four proteins with the *cationic amino acid transporter, C-terminal* domain. The three domain annotations were mutually exclusive. The remainder of the dataset was annotated with domains or families that occurred only once. None of the amino acid transporters in the dataset were annotated as MFS transporters.

When analyzing the dataset, we found that a glutamate-gated, monoatomic ion channel (AtGLR3.3) ended up in our dataset. This was caused by a faulty relation in GO, which listed glutamate-gated ion channels (GO:0004970) as a sub-type of amino acid transmembrane transporters (GO:0015171), contradicting the respective definitions of the terms. This connection was added in 2023, meaning that it did not influence our previous studies, as they used data from 2022. We kept the protein in the dataset to test the performance of training datasets created with our pipeline, and tested the impact of removing it when evaluating the automatic outlier detection method.

#### 2.1.2. Feature Comparison

We calculated the features for all proteins in the dataset, and subsequently trained and evaluated SVM models in a nested 5-fold cross-validation approach. Due to the low sample count in the test set, we decided to shuffle the dataset five times, and repeated the evaluation procedure for each shuffle. The distribution of the scores was then calculated from all 5*5 scores on the respective test sets.

The hyperparameters of the pipeline, including the regularization parameter *C* of the SVM and the number *k* of features to select according to ANOVA F-scores, were optimized by calculating balanced accuracy on the training set. The maximum number of features that could be selected for each model was set to 200, in order to improve the generalization, and *C* was selected as one of three values (C∈{0.1,1.10}). For the test set of each split, the F1 scores of the individual classes were calculated, in order to test for classification performance and class imbalance. The balanced accuracy and macro-averaged F1 score were also calculated as general performance measures across both classes.

**Figure 1:**
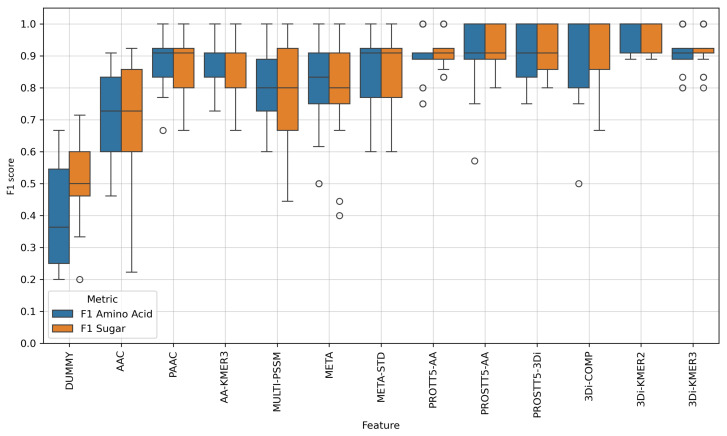
Evaluation results for an SVM model that distinguished sugar from amino acid transporters in *A. thaliana*. This plot visualizes the distribution of F1 scores for each class after five shuffled repetitions of a nested 5-fold cross-validation. Each box represents the 25th to 75th percentile, the line in the box is the median, and dots are test cases that were classified as outliers. A score below the median is determined to be an outlier if the distance between the 25th percentile and the score is greater than the distance between the 25th and 75th percentiles multiplied by 1.5. For scores above the median, the distance to the 75th percentile is used instead. Table A3 provides a short overview of the features, in terms of the data that was used to calculate them. Due to the number of available samples for evaluation, the size of each test set was relatively small (10–11 samples), meaning that the median score calculated from the 25 splits is more representative of real-world performance than the variance. Both the embeddings and the features derived directly from the 3Di sequence outperformed previous approaches, which were based on evolutionary information and amino acid k-mer frequencies.

The means and standard deviations for the features are shown in Table A1, while Figure 1 visualizes their distribution. Since our dataset only contained 54 samples in total, a single false positive or false negative could have a large effect on the resulting score distribution. As there are only 10 or 11 samples in each test set, the accuracy score would increase in steps of 9–10%. We repeated the CV five times to obtain more accurate median scores, whereas the variance might not be representative of the actual performance.

The performance of the dummy feature was as expected for numbers drawn from a uniform random distribution, which confirms that the results are not caused by overfitting or similar issues, and that the remaining features provide additional information compared to randomly drawn values. The slightly higher F1 score for sugar transporters can be traced back to the higher class homogeneity, as all sugar transporters belong to two families, while the proteins in the amino acid class belong to a larger number of families, and therefore potentially have more than two evolutionary ancestors. Both classes had medium F1 scores of 0.5 or lower, and the balanced accuracy was 0.463.

Next, we tested the previously best-performing features for membrane transporter substrate prediction [17,18]. The simple amino acid composition (AAC) with only 20 positions did not capture enough information, leading to a median score of around 0.71, with a large variance. In contrast, PAAC performed surprisingly well on this particular dataset, possibly due to the fact that most proteins in our dataset contain one of a small number of sequence domains that are associated with their respective substrate specificity. The amino acid 3-mer frequency (AA-KMER3) did not perform better than the 2-mer encoding (i.e., PAAC).

The Multi-PSSM feature led to models that performed reasonably well, with the majority of CV splits producing F1 scores between 0.7 and 0.9. Similarly to the AAC, the variance on the sugar class was much larger than on the amino acid transporters. This effect was reduced to two outliers when combining the Multi-PSSM feature with AAC and PAAC into the META feature. The protein fingerprint feature META-STD, which is standardized along the sample axis, no longer produced outliers with F1 scores below 0.6, and had an improved median score (see Figure 1).

The variance in performance between CV splits was much lower for the amino acid embeddings created with ProtT5, whereby only one split led to an F1 score of 0.75 on the amino acid transporter class. A total of 20 out of 25 scores are located within a window of size 0.05 around the median score of 0.91. In addition to generating amino acid sequence embeddings with the ProtT5 model, we also created embeddings with the bi-lingual ProstT5 model. This encoder–decoder transformer model aims to translate directly between amino acid and 3Di sequences, although we only used it to encode either protein sequences from Uniprot or 3Di sequences generated from predicted protein structures, as protein sequence embeddings or protein structure embeddings, respectively. The score distributions between the two models only differed slightly, although there is one split where the amino acid embeddings did not classify the amino acid transporter correctly, while the 3Di embeddings did (see Figure 1).

Since we already calculated 3Di sequences for all protein sequences in our dataset with data from AlphaFoldDB, we also explored the idea of turning them into k-mer frequency features, similar to AAC and PAAC. These 3Di features worked surprisingly well, even beating the embeddings, both in their mean scores and in their consistency across the cross-validation folds. The 3Di 2-mer frequency “3Di-KMER2” worked especially well, capturing enough information about the proteins to reach a mean balanced accuracy of 0.967, with a standard deviation of only 0.046 across all 25 independent test sets, while also showing minimal class imbalance between the sugar and amino acid carriers. Simply comparing the frequencies of the 20 3Di states between the two protein classes was enough for a balanced accuracy of 0.93±0.1, which is impressive when considering that the resulting feature vector only consists of 20 positions. However, the distributions of F1 scores in Figure 1 suggest that the 2-mer frequencies lead to the most stable model, as there were some edge cases where the performance of 3Di-COMP dropped sharply, and where the 3Di-KMER3 produced outliers in the score distribution. All features were evaluated on the same splits, meaning that the 2-mer frequency managed to classify these cases correctly.

As 3Di k-mer frequencies seemed to work so well for encoding substrate binding affinities of membrane proteins, we tried combining them with the amino acid compositions to see if that additional information improved them further. The performance of these combined features (see COMB features in Table A1) was comparable to the embeddings, while producing a higher score variance and lower mean values than the best-performing 3Di-KMER2 feature.

#### 2.1.3. Automatic Outlier Removal

As mentioned before, our automated dataset creation methods sometimes identified a few outliers in the dataset. This can be caused by faulty connections in the GO graph as we found above, or by biological differences between one protein and the rest of the dataset. For example, the proteome of *E. coli*, a gram-negative bacterium that is surrounded by an inner and an outer cell membrane, includes outer-membrane pores (OMPs) that enable a wide range of nutrients and signaling molecules to enter the periplasmic space, including sugars and amino acids. Having these β-barrel pores in the same training dataset as α-helical solute carriers of those molecules can have a very detrimental effect on model training and classification performance.

**Figure A1:**
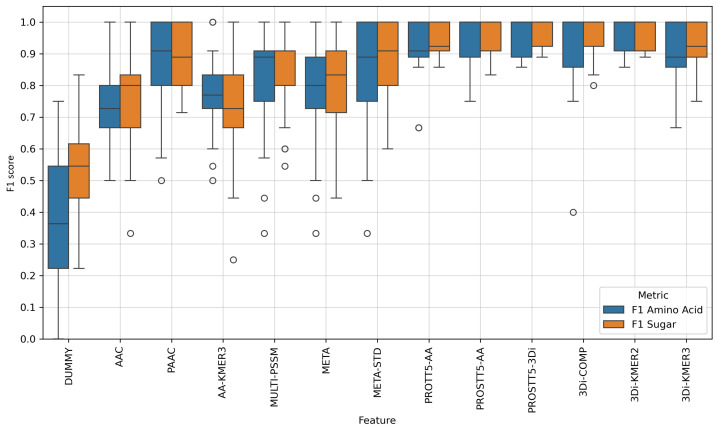
Evaluation results for an SVM model that can distinguish sugar- from amino acid transporters in *A. thaliana*, after automatic outlier removal. This plot visualizes the distribution of F1 scores for each class, after five shuffled repetitions of a nested 5-fold cross-validation. Each box represents the 25th to 75th percentile, the line in the box is the median, and the length of the whiskers is limited to 1.5 times the distance between the 25th and 75th percentile. Scores outside of that range are shown dots, and classified as outliers. In contrast to Figure 1, an automatic outlier detection method has previously been applied to the data, in order to find and remove biological edge cases, and potential errors in the data. As in Figure 1, the 3Di-KMER2 feature delivered the best-performing model. The classification performance of all three embeddings was improved by the outlier removal. The consistency of evaluation scores was also improved, with lower standard deviations for several features.

To automatically handle cases like this, we implemented an automatic outlier detection method using PCA and Isolation Forests (see Section 3.12). This method found the aforementioned glutamate-gated ion channel AtGLR3.3 as an outlier, along with the chloroplast outer-envelope pore protein OEP161, and a lysosome membrane cystine transporter called “Cystinosin homolog”. When enabling the automatic outlier removal during the dataset creation process and repeating the evaluation procedure for our SVM model with all features (see Table A2), we found that the 3Di-KMER2 feature still delivered the best-performing model, with similar mean and standard deviations to the evaluation scores, while the average balanced accuracy for the embeddings improved by 0.11 for the ProstT5-AA feature, and by 0.04 and 0.035 for ProstT5-3Di and ProstT5-AA, respectively. This means that the embeddings generated with the ProstT5 model performed better than the ProtT5 embeddings on the cleaner dataset, while also delivering more consistent results across the 25 test sets (see Figure A1). Another interesting observation is that the COMB-KMER1 feature, which combines the frequencies of 20 amino acid types and 20 3Di states into a feature vector of 40 positions, now led to better results than the 3Di-COMP feature alone, with a higher mean score and lower standard deviation, and produced a much better model than the amino acid composition by itself (see Table A2).

Finally, it should be noted that the outlier removal pipeline was applied to the full dataset before the evaluation, meaning that the evaluation results from this particular test might not reflect the performance on previously unseen data. The purpose of this test was to obtain an estimate of how the model would perform on a perfectly clean dataset, and assess the impact of potential errors introduced by the automated dataset creation pipeline.

#### 2.1.4. Deep Neural Network Model for Membrane Transporter Substrate Prediction

In a previous study [17], we showed that a single-layer perceptron can improve membrane transporter substrate prediction, compared to SVMs. This time, we also wanted to explore how a deep neural network, created with TensorFlow and the Keras API, would compare.

Our DNN model had three hidden layers, with 512, 256, and 128 nodes, respectively. The size of the input layer was adapted for each feature vector (e.g., 1024 for the embeddings, or 400 for 3Di-KMER2). To improve generalization and reduce overfitting, the hidden layers were interspersed with dropout layers that randomly (but deterministically) cut half of the connections during training.

The model was evaluated in a 5-fold stratified cross-validation approach, with five repetitions, where the data was randomly shuffled each time. The same datasets, random seeds, and cross-validation splits were used as for the SVM model, meaning that both models were evaluated on exactly the same training and test splits, without employing outlier removal.

**Figure 2:**
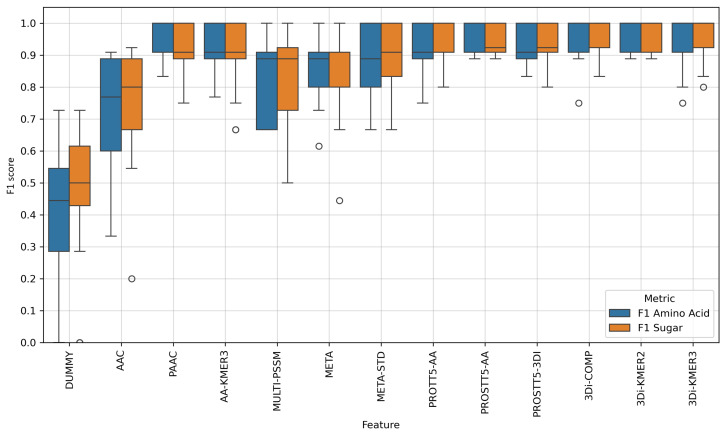
Distribution of 25 test set evaluation scores from five shuffled repetitions of a 5-fold cross-validation when distinguishing sugar transporters from amino acid transporters in *A. thaliana* with a deep neural network (DNN) trained on features that encode the amino acid sequence or protein structure. A short overview of the types of features used is listed in Table A3. The box represents the area between the 25th and 75th percentiles of the score distribution, and has a line in the middle that shows the median. The whiskers extend to 1.5 times the length of the box at most. Any sample below or above the whiskers is classified as an outlier, and shown as a dot. As in Figure 1, the DL-based features in the form of embeddings and 3Di k-mers performed better than the previously developed META-STD feature. Interestingly, the amino acid 2-mer and 3-mers also led to good models when combined with a DNN. Overall, the DNN model produced more consistent results with higher median scores, as well as higher minimum scores for its two best-performing features. Again, the 3Di 2-mers matched the performance of DNNs trained with language embeddings.

The distributions of F1 scores in Figure 2 show a higher consistency across the individual feature datasets, with fewer outliers than the SVM results in Figure 1. This is especially the case for the embeddings and for the 3Di features. The META and PSSM scores delivered improved results, and the embeddings and 3Di compositions achieved high median scores. The best and most consistent results were obtained with the amino acid embeddings from the bi-lingual ProstT5 model, and with the 3Di 2-mer frequencies, which was also the best-performing feature for the SVM model. For both of these features, the lowest F1 score on any class across all 25 independent test sets was 0.89. Interestingly, the results were more consistent than those of the SVM model after outlier removal (as shown in Figure A1), reflecting the common observation that DNNs with multiple layers tend to be less sensitive to outliers than binary SVM models.

When comparing the means and standard deviations of the balanced accuracy scores between the DNN model (Table 1) and the SVM model (Table A1), the same patterns emerge. The 3Di composition gave the highest and most consistent scores, closely followed by the ProstT5 embeddings, and closely behind these were the embeddings from ProtT5. Combining amino acid k-mer frequencies with 3Di k-mer compositions (COMB features) did not improve the results any further.

### 2.2. Categorization of Human Ion Channels

One type of transport protein that previous models struggled to classify was (monoatomic) ion channels. In contrast to the binding pockets that are commonly found in carrier proteins and many primary active transport complexes, these channels employ selectivity filters, i.e., narrow paths with multiple binding sites for ions and often for water molecules. Typically, these ion interaction sites are mainly made up of backbone carbonyl groups, although they are sometimes interspersed by charged residues, e.g., to orient substrate molecules in the channel. The selectivity of these channels is mainly determined by their width, and possibly by their flexibility [59]. This stands in contrast to the binding pockets of carrier proteins, where the residues need to match the chemical properties of atoms in the transported molecule in order to form non-covalent interactions [2]. On the other hand, several families of ion channels possess selectivity filters with conserved sequence domains. Since both ion channels and carrier proteins can contain conserved sequence motifs and conserved structural domains that are directly associated with their substrate specificity [2,59], we explored how well the sequence- and structure-based feature encodings described in this study can capture these differences in ion channels, and how they compare in terms of classification performance.

#### 2.2.1. Dataset Analysis

To estimate how well the new features and models are able to encode the substrate specificity of monoatomic ion channels, we created datasets of all human channel proteins transporting either calcium, chloride, or potassium. GO annotations inferred by electronic annotation were not included in the dataset. Proteins were filtered for those in the manually reviewed Swiss-Prot subset of UniprotKB, which also had evidence for the existence of the protein sequence, and known gene names. Sequence fragments were excluded. After dataset creation, where we only kept experimentally verified data, we were left with 105 calcium channels, 100 potassium channels, and 72 chloride channels, respectively.

According to GO terms annotated with experimental evidence, the channels possess a wide range of gating mechanisms. Out of 105 calcium channels, 36 are known to be voltage-gated, 26 are ligand-gated, 10 are store-operated [60], and 1 is stretch-activated. Only 11 out of 72 chloride channels are voltage-gated, whereas the 40 ligand-gated channels are activated by a wide range of molecules, including calcium (19 channels), GABA (14 channels), glycine (5 channels), and ATP and glutamate, which both activate 1 channel each. Four volume-sensitive and two pH-sensitive Ca^2+^ channels were in the dataset as well. The vast majority of potassium channels are voltage-gated, 78 out of 100 in total. Ligand-gated potassium channels are activated by calcium (11 channels), sodium (2 channels), as well as chloride and ATP, which activate 1 channel each. Further, three channels are mechano-sensitive. This means that potassium channels were the most extensively studied and annotated channel type in our dataset, by a large margin.

The overlaps between the classes were small, with five proteins that carry both Ca^2+^ and K^+^, and one channel that transports Ca^2+^ and Cl^−^ simultaneously. We decided to exclude these proteins from the respective binary classification tasks, as their numbers would be too small to provide enough information to a multi-output model, or to add them as a third class. One channel in each of the three substrate classes did not have a 3D structure available in AlphaFoldDB, and was therefore excluded from the dataset as well.

Finally, we applied a sequence identity threshold of 70% to each substrate class. This left us with three binary classifications tasks: 100 calcium channels and 58 chloride channels, 100 calcium channels and 84 potassium channels, and 88 potassium channels against 59 chloride channels.

In summary, all three substrate classes contained channels from a wide range of families and mechanisms, and from different evolutionary origins. This means that the evaluation of ML models on these datasets reflects their ability to distinguish between the actual substrates that are selected by the central cavity.

#### 2.2.2. Results for SVM and DNN Models

The SVM and DNN models were evaluated on the same datasets, with the same 25 cross-validation splits. This was achieved by repeating a 5-fold cross-validation five times, shuffling the dataset after each CV, and starting with the same random seed. The feature datasets were exported from the SVM evaluation after all filtering was finished, and subsequently imported directly into the DNN evaluation methods.

**Figure 3:**
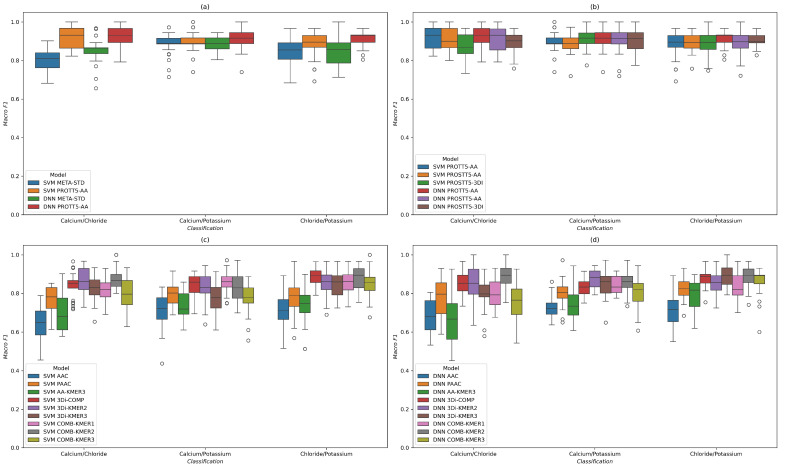
Distribution of macro-averaged F1 scores across five shuffled repetitions of a 5-fold cross-validation. Either a support vector machine (SVM) with feature selection or a deep neural network (DNN) were trained to distinguish between human ion channels in binary classification tasks. All tests were carried out on the same splits of the same datasets. The scores were calculated on the test set of each CV split. The boxes show the area between the 25th and 75th percentiles, and the whiskers extend in length to 1.5 times the range between these percentiles at most. Any score that is further away is visualized as a dot, denoting an outlier. The features are categorized according to feature generation methods and underlying data in Table A3. In (**a**), we compared our previously developed META-STD feature to amino acid sequence embeddings calculated with ProtT5, and also tested the impact of replacing the SVM model with a DNN. The differences between two different amino acid embeddings and a 3D structure embedding are shown in (**b**). While the DNN model improved the median and minimum scores for several features, the choice of feature generation algorithm had a larger impact on model performance. In (**c**,**d**) we compare all features derived from k-mer frequencies in the protein sequence and/or the 3Di structure sequence with an SVM and DNN model, respectively. Each classification task seems to have a different combination of features and model that leads to the best performance, suggesting that an ensemble method would likely be necessary in order to compete with the performance of protein language embeddings.

First, we compared the previously best-performing model, namely, an SVM model with ANOVA F-score feature selection and META-STD protein fingerprint feature, to a DNN trained on the same feature, as well as an SVM and a DNN trained on amino acid embeddings (see Figure 3a). While switching the model to a deep neural network increased the median score for Ca^2+^/Cl^−^, as well as the maximum and minimum score for the other two tests, a larger effect came from the new feature, which improved minimum scores in all cases and median scores in most. After replacing the META-STD feature with ProtT5 embeddings, the mean and standard deviation of the SVM model were improved by 0.07, while the standard deviation was lowered by 0.01 when distinguishing between calcium channels and chloride channels (see Table 2).

Next, we explored how the additional amino acid and 3D-structure embeddings we encoded with the ProstT5 model performed in comparison to the ProtT5 embeddings, both with the SVM pipeline and with the deep learning model (see Figure 3b). Overall, the performance of these features on ion channels was quite similar. In the Ca^2+^/Cl^−^ classification, the 3Di features led to lower median scores, whereas DNN led to slight improvements compared to the SVM. For Ca^2+^/K^+^, the 3Di embedding feature managed to improve the scores of the SVM model, delivering very similar results to the DNN model with amino acid embeddings. The best minimum score and lowest standard deviation were observed for the 3Di/DNN model for distinguishing between chloride channels and potassium channels, whereas the best median score came from the DNN with ProtT5 embeddings. According to the mean and standard deviations in Table 2, the embedding models led to the best performance in most cases, with the META-STD and 3Di frequency features often close behind.

Furthermore, we explored how the k-mer frequency features, which can be directly calculated from amino acid or 3Di sequences, compared to each other. For this comparison, we trained an SVM model, as shown in Figure 3c, and a DNN model, shown in Figure 3d, again on the same 25 train/test splits that were used for all four plots in Figure 3. The noteworthy point is that the amino acid k-mer frequencies delivered the lowest performance across all models and classification tasks, with only the PAAC feature, which contains 2-mer frequencies, delivering adequate results. This was expected, as it was the reason why we combined the AAC and PAAC with Multi-PSSM into the META-STD feature, which led to better-performing models than its separate parts (as can also be observed in Table 2). Table 2 also shows that PSSMs calculated from more distantly related proteins, e.g., those found by three iterations of PSIBLAST on Uniref50, consistently led to better-performing features than PSSMs derived from closer homologues, such as those found with one iteration on Uniref90, suggesting a possible connection to model performance. The 3Di sequence features gave better predictive models in all three cases when paired with either of the two ML algorithms, similarly to what we observed when categorizing *A. thaliana* carrier proteins in Section 2.1.2. Combining the information from both sequence types led to better results in most tests, especially when combining PAAC with 3Di 2-mers (COMB-KMER2), which was among the best features across all tests and for both the SVM and DNN model.

Again, we found that the difference lies mainly in the choice of features and less so in the model itself, at least when comparing the SVM and DNN models from this study. The other consistent observation was that each combination of substrate classes prefers a particular type of feature over another. Therefore, a purely sequence-based method that is competitive with protein embeddings will likely have to be an ensemble approach that creates different models from both amino acid and structural sequences, and then selects the best one based on training data performance.

## 3. Methods

### 3.1. Data Availability

All code used in this study is provided in a git repository at https://github.com/adenger/subpred_dl (accessed on 28 July 2025), together with a reproducible coding environment that contains all dependencies. Links to archives containing raw and pre-processed data can be found in the description of the repository. Together with the code and environment, the results found in this study can be fully recreated. The “preprocessing” folder contains all scripts that were used to download and process the raw data.

### 3.2. Protein Dataset Creation

Annotated protein datasets were automatically assembled with a dataset creation pipeline. Protein sequence data was retrieved programmatically from the Uniprot [61] API (version 2025_02). This table contains all proteins from Swiss-Prot (reviewed) and TrEMBL (unreviewed), along with accessions from other databases, such as Uniprot Keywords, Interpro [58], gene names, and Transporter Classification Database (TCDB) [62] identifiers. Only proteins with non-fragmented sequences were retrieved. We only kept proteins in the dataset where the existence of the protein sequence had been experimentally verified at the protein level, and where the encoding gene was known.

As some of the machine learning features used in this study were calculated on predicted 3D structures from AlphaFoldDB [28,48], which does not consider proteins with non-standard amino acid codes such as B, O, U, Z, and X, we removed these proteins from our datasets.

The code for the dataset creation pipeline was adapted from our previous studies on transmembrane transporter substrate prediction [17,18], and can now create datasets for any task that involves the prediction of GO terms for sets of protein sequences or 3D structures, not just transporter datasets.

Sequence clustering was carried out on the dataset with CD-HIT [63] to prevent two very similar proteins from the same class being part of the training and test sets. As in previous studies, we employed a threshold of 70% sequence identity [17].

### 3.3. Gene Ontology Graph and GO Annotations

Functional annotations about substrate specificity were retrieved as Gene Ontology [64] (GO) terms from the *molecular function* aspect of the Gene Ontology. The full dataset of Gene Ontology annotations [65] for Uniprot (GOA-Uniprot-All version 226) was downloaded from the FTP server of the European Bioinformatics Institute (EBI) in the GO annotation format (GAF, version 2.2), and subsequently filtered for relevant columns and rows using AWK (version 5.3.1) and Python (version 3.12.10). During filtering, only annotations of Uniprot accessions were kept, along with information about the evidence quality, and about the type of relation between each protein and GO term. In a final pre-processing step, all but the proteins from Section 3.2 were removed, reducing the compressed file size from 21 GB to 280 MB.

Relations between the GO terms were downloaded from the official GO website, in the OBO format [66] (database version 2025-03-16). They were read into a NetworkX (version 3.4.2) [67] graph data structure using the obonet Python package (version 1.1.1).

When a machine learning protein dataset with GO annotations was created with specified parameters, the implicit GO annotations for all proteins were derived from the explicitly listed annotations and added to the dataset, using graph search methods from the NetworkX package. When a protein was annotated with a specific GO term, and that GO term was connected to more abstract GO terms through the “is_a” relation, then the protein was annotated with those ancestor terms as well.

### 3.4. Blast Databases and PSSM Calculation

Position-specific scoring matrices (PSSMs) were calculated with PSI-BLAST [68] on local protein-BLAST databases created from Uniref50 and Uniref90 (version 2022_05) [69]. Both the PSSMs and the BLAST databases were generated with the blast+ package (version 2.16.0) [70]. We calculated PSSMs for each protein with either one or three iterations of PSI-BLAST on either of the two BLAST databases, with an E-value threshold of 0.002. PSSMs were stored in a cache folder, which greatly reduces the runtime of subsequent runs. A script for updating the BLAST database is provided in the repository.

### 3.5. Predicted 3D Structures and 3Di Sequences

Predicted 3D structures were downloaded from the AlphaFoldDB (version 4) [28,48] FTP server for four model organisms: *A. thaliana*, *E. coli*, *S. cerevisiae*, and human.

The 3D structures of all proteins in the AlphaFoldDB archives were converted to 3Di sequences [54] using the Foldseek command line program (version 10.941cd33). To this end, a custom Foldseek database was created from all extracted PDB files, and subsequently converted to a Fasta file containing the 3Di representations.

Human proteins with a sequence length of more than 2700 amino acids were split into multiple, overlapping PDB files by AlphaFoldDB, where each subsequence (usually except for the last one) was 1400 amino acids long and had an overlap of 1200 positions with the next substructure. We created the 3Di sequences for each of these structure fragments separately, and then merged the 3Di sequences back into a single sequence by starting with the first sequence and subsequently adding the suffix after position 1200 from each of the following splits (see Section 2.1.1 for statistics).

### 3.6. Machine Learning Features Derived from Amino Acid Sequence

Amino acid sequences were encoded using three different feature generation algorithms that turn variable-length amino acid sequences into fixed-length numerical vectors.

The amino acid composition (AAC) is the frequency of each amino acid type in the standard genetic code, divided by the length of the sequence. The pair amino acid composition (PAAC) is the frequency of each possible pair of two amino acids, divided by the total number of pairs in the sequence (i.e., its length minus one). We also created a tri-peptide composition from the relative frequencies of all possible subsequences of length 3.

The PSSM feature was derived from a position-specific scoring matrix. For a sequence of length *n*, this PSSM is a n×20 integer matrix, containing the log-odds scores of exchanging the amino acid type at any position *i* (1≤i≤n) with any of the 20 amino acid types throughout evolution.

For all sequence positions with a particular amino acid type, the log-odds vectors at those positions are added together along the sequence axis, and divided by the number of occurrences of that amino acid type in the sequence to calculate the average vector. The resulting 20×20 matrix reflects the evolutionary conservation profile of the protein.

Finally, the features were combined in different ways. As the PSSM matrices for each protein all contained log-odds values, we concatenated their rows into one Multi-PSSM feature that includes data from closely and distantly related proteins, due to different choices of BLAST databases and PSI-BLAST parameters for each feature matrix. In a previous study [17], we created the META-feature from AAC, PAAC, and multiple PSSMs by standardizing each feature along the sample axis and then concatenating the standardized vectors. This avoids information sharing as each sample is standardized independently of the others, but can change the scales along the feature dimensions, meaning that the META-feature should be seen as a fingerprint of the amino acid sequence.

As most of these features had more dimensions than we had samples in our dataset, we employed feature selection, dimensionality reduction and regularization techniques, along with a sound evaluation procedure, to prevent and test for overfitting.

### 3.7. Machine Learning Features Derived from 3Di Sequence

The 3Di sequences can be used as input for many methods that were originally invented for amino acid sequences, as similar structures lead to similar sequences (where the similarity can be calculated with a provided substitution matrix). Therefore, we applied AAC, PAAC, and KMER3 feature generation to these structural sequences as well, resulting in the “3Di_COMP”, “3Di_KMER2”, and “3Di_KMER3” features, respectively.

Finally, the information from amino acid and structural sequences were combined by concatenating AA and 3Di k-mer frequencies of the same lengths into the “COMB_KMER1”, “COMB_KMER2”, and “COMB_KMER3” features.

### 3.8. Embeddings of Protein Sequence and Structure

The proteins sequences were encoded with two different Text-To-Text Transfer Transformer (T5) protein language models (pLMs) [57]. Converting a sequence through the provided tokenizer and using it as input for the model resulted in a 1024-dimensional embedding for each token, i.e., a numerical vector of length 1024 for each position in the input sequence. These per-residue embeddings were then turned into per-protein representations with mean-pooling, where the average embedding for each type of token (e.g., each amino acid type) was calculated.

Pre-trained T5 models were retrieved through the Huggingface transformers API [71], and subsequently used to generate protein embeddings locally. The ProtT5 model [23] was used to encode amino acid sequences, resulting in a 1024-dimensional numerical feature vector for each protein in the dataset after mean-pooling. Furthermore, we employed the encoder of the bilingual ProstT5 model [56], which aims to translate between AA and 3Di sequences directly, to encode both AA and 3Di sequences. To this end, we used the corresponding T5 encoder, along with the provided tokenizer, to encode either 3Di sequences or AA sequences into mean-pooled per-protein embeddings.

### 3.9. Dummy Feature

We also added a “DUMMY” feature in each test, which consisted of 1024 positions that were deterministically drawn from a uniform random distribution with numpy [72]. The purpose of this non-informative feature was to estimate the amount of additional information that is provided by the real features calculated from the proteins, and how much more stable the resulting models are. A high score of the DUMMY feature can be a sign that the model is overfitting, as this can cause the model to find non-existent patterns in the data.

### 3.10. Support Vector Machine (SVM) Models

All tasks related to training and optimizing the SVM model pipeline were carried out with scikit-learn (version 1.6.1) [73]. The SVM models [74] used an RBF kernel. The corresponding regularization parameter γ, where smaller values reduce the influence of individual samples on the decision boundary, was automatically scaled inversely to the number of features and the variance in the training data, as

$$\gamma =\frac{1}{var(X_{train})\cdot m},$$

where *m* is the number of features and $X_{train}$ is the training split of the feature dataset.

The value of *C*, which determines the penalty for samples located inside the decision boundary, was optimized between three values through hyperparameter optimization (C∈{0.1,1,10}). *C* was multiplied with different class weights for each class *i*, calculated as

$$weight_{i}=\frac{n}{K*n_{i}},$$

where *n* denotes the total number of samples, *K* is the number of classes, and $n_{i}$ represents the number of samples annotated with class *i*.

Feature selection and pre-processing were carried out in three steps. To avoid information sharing, all constants and scores were derived from the training set and only applied to the test set. First, all features with a variance of exactly 0 in the training data were removed. This is especially important for the KMER3 features, as there are $20^{3}=8000$ possible 3-mers for an alphabet size of 20, many of which did not appear in any protein. For each of the remaining features, ANOVA F-scores were calculated based on the labeled training data, and the *k* features with the highest score (i.e., those that showed the highest variance between classes relative to the variance within the classes) were selected. The number *k* was optimized through hyperparameter optimization, and was limited to $k\leq min(200,m^{\prime })$, where $m^{\prime }$ is the number of feature dimensions left in the dataset after initially applying the variance threshold. Finally, the selected features were standardized by calculating z-scores along the feature axis, with mean and standard deviation derived from the training split.

The hyperparameter optimization was carried out through a grid search, where each combination of parameters was tested in a 5-fold stratified cross-validation on the training split. To obtain an even performance between both classes, the hyperparameters were optimized with the balanced accuracy score.

### 3.11. Deep Neural Network (DNN) Model

Deep neural networks (DNNs) for binary classification of substrates were created with TensorFlow (version 2.18.0) [75] using the Keras [76] Sequential API. The shape of the input layer was matched to the dimensions of each feature. This was followed by three densely connected hidden layers using the ReLU activation function and containing 512, 256, and 128 nodes, interspersed with dropout layers that aim to prevent overfitting and improve generalization by randomly cutting 50% of the connections during training. Finally, a single output node with a sigmoid activation function provided the predicted probability score p∈[0,1] that a given sample was a member of the positive class. During inference, a sample is assigned to the positive class if p>0.5, otherwise it was predicted as negative.

The model was optimized with the Adaptive Moment Estimation (Adam) [77] optimizer, using binary cross-entropy as the loss function. To account for the low sample count in our datasets, we trained the model using a batch size of eight samples and limited the number of training epochs to 50.

### 3.12. Automatic Outlier Detection

After the feature generation was finished, the individual feature datasets were first submitted to an outlier detection pipeline. After standardization, each feature dataset was converted to a number of principal components that explain at least 95% of variance in the data, using principal component analysis (PCA). Next, an Isolation Forest [78] assigned an anomaly score to each sample and used that, together with an automatically determined anomaly threshold, to classify samples as outliers. If a sample was found to be an outlier in at least 50% of feature datasets, it was flagged as an outlier for further analysis and can optionally be removed. As this detection was performed using the entire dataset, we excluded this step when evaluating models with cross-validation.

### 3.13. Evaluation Methods

Evaluation of the SVM model was carried out in a nested, stratified 5-fold cross-validation (5FCV) with five repetitions. For the outer cross-validation, the dataset was split into five parts of similar size. Each of the five subsets was used once for validation, while the remaining four were combined into a training set. On this training set, internal 5FCV’s were carried out with every combination of parameters, and the best set of parameters across all five splits was selected. The model was then fitted to the entire training dataset with the best parameters, and tested on the validation set. As the validation sets can be relatively small, an additional true positive can have a considerable impact on the score. To reduce the impact of this stepping on the results, we shuffled the dataset five times in a deterministic fashion and repeated the outer 5FCV for each of the shuffled datasets. The final evaluation score was then calculated from all 5 × 5 = 25 test scores from the evaluation sets. Stratified splits ensured that the ratio between the classes remained as close to the full dataset as possible.

As both classes had equal importance and some of the datasets showed a class imbalance, the hyperparameter optimization was carried out with the balanced accuracy score, i.e., the average recall score across all classes. To detect a class imbalance between the two substrate classes, an F1 score was calculated for each class individually. As the F1 score is the harmonic mean between precision and recall, a high F1 score on both classes means that both precision and recall for either substrate class are sufficient. Finally, we estimated overall performance through the balanced accuracy score and macro-averaged F1 scores. These metrics are calculated as the arithmetic mean between the performance on the individual classes, meaning that lower scores have to be interpreted more carefully.

The DNN model was evaluated on the same training and test sets that were used for the outer loop during the SVM evaluation, but did not require the inner loop as we did not adjust any hyperparameters based on training data. Regularization was instead carried out by randomly deactivating connections during training.

## 4. Discussion

In this study, we compared our previously developed methods for membrane transporter substrate prediction [17,18], where we trained support vector machines (SVMs) on ML-features derived from amino acid sequence and evolutionary information, against new feature extraction and classification techniques empowered by advancements in deep learning and natural language processing.

We used the encoder from the pre-trained ProtT5 transformer model to turn amino acid sequences into fixed-size word embeddings, which were then averaged across the protein sequence to create a numerical representation. Furthermore, we retrieved 3D structural models for the considered protein sequences that were predicted by the AlphaFold transformer model, and then used Foldseek to turn the three-dimensional atomic configurations into 3Di sequences, which represent protein structures with an alphabet of 20 characters. These 3D-structure sequences were then turned into embeddings with the bi-lingual ProstT5 model that has been trained on both types of sequences, and which we also used to generate a second dataset of amino acid embeddings. We also directly calculated ML-features from the 3Di sequences by turning them into vectors of k-mer frequencies, and also combined those with k-mer frequencies of amino acids. Finally, we trained our own DNN with TensorFlow, and compared the performance to our previously developed SVM model. To estimate the amount of information that is provided by the features, we also included a feature that was drawn from a uniform random distribution.

We tested our models by predicting the substrates of carrier proteins in *A. thaliana*, and of ion channels in human, in a cross-validation approach. When analyzing the dataset of sugar and amino acid transporters in *A. thaliana*, we found that each substrate class contained proteins from multiple protein (super-)families with no or little evolutionary relation to each other. This indicates that our models were in fact predicting the substrate, and not, for example, the superfamily fold.

When training and evaluating an SVM on the data, the feature made up of 3Di k-mer frequencies surprisingly outperformed the protein embeddings, both in the mean score and the standard deviation across the 25 test sets. The 3Di 2-mer frequencies delivered particularly good results, with a mean balanced accuracy of 0.971±0.044, which was 0.061 higher than the ProtT5 amino acid embeddings (see Table A1), as well as the highest minimum score (Figure 1). Combining frequencies of amino acid k-mers with the 3Di features produced similar results as the three embeddings, of around 0.90.

Our automatic outlier detection method classified three amino acid transporters as divergent from the rest of the data, which made sense upon further inspection. Removing these three proteins allowed us to test the performance on a “clean” dataset. This improved the average evaluation scores of the ProstT5 models compared to the ProtT5 embeddings (Table A2), bringing the performance of the 3Di embeddings close to that of the 3Di 2-mer frequencies (Figure A1).

Finally, we tested the TensorFlow DNN model on the automatically generated dataset (i.e., without removing any outliers), and found improvements to most features in terms of their average performance, as well as their consistency across the different training and test sets. Again, the 3Di 2-mer model led to the best performance, with a balanced accuracy of 0.959±0.047, closely followed by the ProstT5 embeddings, and the combined 3Di and amino acid frequencies, with better mean and standard deviation compared to the ProtT5 embeddings (Table 1). Both the ProstT5-AA and 3Di 2-mer models delivered very stable models, with the lowest observed test scores around 0.89 (Figure 2).

For the task of distinguishing three types of human ion channels in three binary classifications, the same three features delivered the best results, but their evaluation scores were arranged in the opposite order. This time the embeddings led to mean macro-F1 scores between 0.90 and 0.92, followed by combined PAAC and 3Di 2-mers features of around 0.84–0.89, and finally the 3Di 2-mer feature on its own, falling between 0.83 and 0.88 (Table 2). Replacing the SVM with a DNN led to relatively small differences in the mean and standard deviation of the scores across the 25 tests, while the bigger difference came from the choice of the features themselves (Figure 3). As for the *A. thaliana* dataset, we analyzed the protein families and co-occurring GO terms in the dataset, and found multiple different gating mechanisms and protein families in each of the three substrate classes.

An interesting aspect when considering the performance of the 3Di frequencies is the time it takes to calculate each feature. Deriving a single PSSM profile from Uniref50 or Uniref90 with PSI-BLAST can take several minutes, even on a fast compute server, and the Multi-PSSM feature (and therefore also the META-feature) requires four PSSMs for each protein. Calculating protein embeddings locally requires a similar amount of time when using the CPU, but the process can be accelerated to less than a minute per protein with a CUDA-compatible graphics card and a sufficient amount of available video memory. On the other hand, the 3Di sequences for an entire proteome can be calculated with Foldseek in less than one second, and the only requirement is a database of protein structure files, which can be created or downloaded through the same tool. The 3Di k-mer feature could therefore be a faster alternative to embeddings or to features that encode evolutionary conservation, e.g., for large-scale analyses.

In conclusion, we found that deep learning delivers the necessary tools to both encode and accurately predict the substrate specificity of membrane transporters, improving on existing approaches such as SVMs trained on amino acid frequencies and evolutionary conservation profiles. However, the exact ML model and feature required depend on the substrates in question. This is due to the fact that transporters of one substrate can include many different protein families, domains, and 3D folds, with different or common evolutionary origins. Finding a model that captures the exact information needed to determine the substrate specificity while also tuning out confounding variables, such as a common evolutionary origin between some proteins, likely requires an ensemble approach that selects a model and a type of feature based on training data. Furthermore, combining data from several organisms, either directly or through transfer learning, could allow for the method to be applied to datasets with an insufficient number of training samples.

## Figures and Tables

**Table 1:** Mean and standard deviation of 25 evaluation scores from five shuffled repetitions of a 5-fold cross-validation when distinguishing sugar transporters from amino acid transporters in *A. thaliana* with a deep neural network (DNN) and different features derived from the amino acid sequence or protein structure. The best results are underlined.

Feature	Balanced Accuracy	F1 Amino Acid	F1 Sugar
DUMMY	0.475 ± 0.120	0.400 ± 0.216	0.477 ± 0.207
AAC	0.754 ± 0.155	0.742 ± 0.156	0.754 ± 0.171
PAAC	0.925 ± 0.061	0.926 ± 0.059	0.915 ± 0.073
AA-KMER3	0.915 ± 0.080	0.919 ± 0.075	0.899 ± 0.103
MULTI-PSSM	0.815 ± 0.122	0.807 ± 0.121	0.813 ± 0.141
META	0.859 ± 0.099	0.855 ± 0.093	0.851 ± 0.123
META-STD	0.889 ± 0.091	0.883 ± 0.097	0.890 ± 0.096
COMB-KMER1	0.917 ± 0.084	0.909 ± 0.100	0.915 ± 0.085
COMB-KMER2	0.945 ± 0.057	0.945 ± 0.058	0.942 ± 0.064
COMB-KMER3	0.945 ± 0.052	0.947 ± 0.051	0.940 ± 0.058
PROTT5-AA	0.918 ± 0.063	0.910 ± 0.071	0.923 ± 0.058
PROSTT5-AA	0.944 ± 0.047	0.941 ± 0.050	0.946 ± 0.046
PROSTT5-3DI	0.937 ± 0.051	0.933 ± 0.053	0.938 ± 0.053
3Di-COMP	0.959 ± 0.056	0.953 ± 0.065	0.963 ± 0.049
3Di-KMER2	0.959 ± 0.047	0.958 ± 0.049	0.959 ± 0.048
3Di-KMER3	0.959 ± 0.067	0.955 ± 0.074	0.963 ± 0.061

**Table 2:** Means and standard deviations of macro-averaged F1 scores across five shuffled repetitions of a 5-fold cross-validation, which led to 25 test set scores in total for each test. Both the deep neural network (DNN) and the support vector machine (SVM) model were tested across three classification tasks, where the goal was to distinguish between two classes of human ion channels. All three substrates are shortened to their chemical symbols (Ca = calcium, Cl = chloride, K = potassium). The dummy feature consisted of 1024 random numbers drawn from a uniform random distribution for each protein, and showed the performance of a feature with no useful information. The best mean and standard deviation for each test are underlined.

Model	DNN			SVM		
Substrate	Ca/Cl	Ca/K	Cl/K	Ca/Cl	Ca/K	Cl/K
Feature						
DUMMY	0.522 ± 0.088	0.477 ± 0.062	0.498 ± 0.088	0.459 ± 0.084	0.505 ± 0.080	0.471 ± 0.078
AAC	0.684 ± 0.087	0.725 ± 0.053	0.712 ± 0.088	0.645 ± 0.084	0.703 ± 0.086	0.705 ± 0.081
PAAC	0.775 ± 0.094	0.805 ± 0.075	0.828 ± 0.063	0.767 ± 0.067	0.795 ± 0.059	0.787 ± 0.083
AA-KMER3	0.663 ± 0.122	0.752 ± 0.080	0.798 ± 0.078	0.700 ± 0.097	0.743 ± 0.076	0.741 ± 0.077
3Di-COMP	0.844 ± 0.060	0.831 ± 0.044	0.876 ± 0.050	0.839 ± 0.068	0.832 ± 0.055	0.885 ± 0.043
3Di-KMER2	0.854 ± 0.082	0.877 ± 0.049	0.845 ± 0.058	0.860 ± 0.068	0.832 ± 0.075	0.854 ± 0.065
3Di-KMER3	0.795 ± 0.078	0.855 ± 0.076	0.894 ± 0.060	0.825 ± 0.066	0.777 ± 0.076	0.845 ± 0.061
COMB-KMER1	0.798 ± 0.077	0.848 ± 0.049	0.837 ± 0.071	0.817 ± 0.063	0.857 ± 0.063	0.864 ± 0.061
COMB-KMER2	0.889 ± 0.063	0.861 ± 0.054	0.882 ± 0.059	0.880 ± 0.056	0.837 ± 0.066	0.880 ± 0.060
COMB-KMER3	0.755 ± 0.095	0.801 ± 0.080	0.861 ± 0.076	0.798 ± 0.082	0.773 ± 0.079	0.847 ± 0.058
PSSM-50-1	0.769 ± 0.084	0.834 ± 0.067	0.791 ± 0.071	0.755 ± 0.061	0.808 ± 0.063	0.783 ± 0.071
PSSM-50-3	0.796 ± 0.052	0.863 ± 0.061	0.811 ± 0.075	0.803 ± 0.072	0.839 ± 0.056	0.806 ± 0.069
PSSM-90-1	0.769 ± 0.105	0.776 ± 0.050	0.778 ± 0.083	0.739 ± 0.070	0.742 ± 0.045	0.759 ± 0.067
PSSM-90-3	0.784 ± 0.084	0.773 ± 0.062	0.781 ± 0.087	0.739 ± 0.070	0.742 ± 0.045	0.759 ± 0.067
MULTI-PSSM	0.780 ± 0.077	0.833 ± 0.072	0.785 ± 0.082	0.783 ± 0.057	0.797 ± 0.052	0.770 ± 0.077
META	0.782 ± 0.072	0.803 ± 0.084	0.812 ± 0.065	0.826 ± 0.062	0.812 ± 0.061	0.784 ± 0.082
META-STD	0.851 ± 0.069	0.891 ± 0.038	0.850 ± 0.080	0.807 ± 0.055	0.884 ± 0.061	0.839 ± 0.072
PROTT5-AA	0.921 ± 0.059	0.915 ± 0.056	0.918 ± 0.043	0.922 ± 0.058	0.899 ± 0.054	0.889 ± 0.056
PROSTT5-AA	0.909 ± 0.065	0.903 ± 0.065	0.898 ± 0.061	0.911 ± 0.062	0.893 ± 0.054	0.898 ± 0.045
PROSTT5-3DI	0.894 ± 0.057	0.908 ± 0.053	0.908 ± 0.038	0.878 ± 0.068	0.911 ± 0.050	0.890 ± 0.061

## Data Availability

The original data presented in the study are openly available at https://github.com/adenger/subpred_dl (accessed on 20 July 2025).

## Appendix A

**Table A1:** Evaluation results for each feature after a nested cross-validation that was repeated five times on shuffled data. For each split, an SVM model was trained to distinguish between sugar-amino acid transporters in *A. thaliana*. The scores represent the mean and standard deviation of the independent test sets, across all 25 splits. The underlying protein dataset was automatically generated from Uniprot and annotated with GO terms, in a way that relies on experimental data as much as possible, while filtering out predicted annotations and proteins. The best mean and standard deviation for each column is underlined.

Feature	Balanced Accuracy	F1 Amino Acid	F1 Sugar
DUMMY	0.463 ± 0.122	0.393 ± 0.171	0.509 ± 0.118
AAC	0.711 ± 0.159	0.705 ± 0.152	0.700 ± 0.202
PAAC	0.892 ± 0.087	0.893 ± 0.086	0.881 ± 0.099
AA-KMER3	0.883 ± 0.082	0.883 ± 0.080	0.870 ± 0.099
MULTI-PSSM	0.816 ± 0.122	0.815 ± 0.118	0.799 ± 0.153
META	0.818 ± 0.134	0.813 ± 0.139	0.808 ± 0.153
META-STD	0.869 ± 0.125	0.866 ± 0.129	0.864 ± 0.128
COMB-KMER1	0.897 ± 0.082	0.894 ± 0.084	0.894 ± 0.088
COMB-KMER2	0.907 ± 0.079	0.908 ± 0.076	0.898 ± 0.092
COMB-KMER3	0.947 ± 0.072	0.947 ± 0.072	0.942 ± 0.078
PROTT5-AA	0.910 ± 0.058	0.900 ± 0.070	0.917 ± 0.049
PROSTT5-AA	0.913 ± 0.079	0.902 ± 0.102	0.921 ± 0.064
PROSTT5-3Di	0.909 ± 0.074	0.899 ± 0.087	0.917 ± 0.067
3Di-COMP	0.929 ± 0.106	0.917 ± 0.127	0.936 ± 0.091
3Di-KMER2	0.971 ± 0.044	0.969 ± 0.046	0.970 ± 0.045
3Di-KMER3	0.920 ± 0.054	0.916 ± 0.058	0.920 ± 0.055

## Appendix C

**Table A2:** Evaluation results for each feature, after a nested cross-validation that was repeated five times on shuffled data. For each split, a SVM model was trained to distinguish between sugar and amino acid transporters in *A. thaliana*. The scores represent the means and standard deviations of the independent test sets across all 25 splits. The underlying protein dataset was automatically generated from Uniprot and annotated with GO terms, in a way that relies on experimental data as much as possible, while filtering out predicted annotations and proteins. In contrast to Table A1, an outlier prediction pipeline was applied to the dataset before the evaluation, which removed three proteins. The best mean and standard deviation for each column is underlined.

Feature	Balanced Accuracy	F1 Amino Acid	F1 Sugar
DUMMY	0.480 ± 0.136	0.354 ± 0.232	0.534 ± 0.156
AAC	0.766 ± 0.116	0.731 ± 0.139	0.770 ± 0.147
PAAC	0.881 ± 0.105	0.864 ± 0.134	0.879 ± 0.095
AA-KMER3	0.770 ± 0.133	0.774 ± 0.126	0.727 ± 0.171
MULTI-PSSM	0.842 ± 0.138	0.812 ± 0.173	0.847 ± 0.133
META	0.810 ± 0.154	0.783 ± 0.181	0.813 ± 0.153
META-STD	0.869 ± 0.135	0.844 ± 0.176	0.876 ± 0.126
COMB-KMER1	0.951 ± 0.080	0.945 ± 0.091	0.951 ± 0.086
COMB-KMER2	0.938 ± 0.083	0.934 ± 0.086	0.933 ± 0.092
COMB-KMER3	0.914 ± 0.088	0.906 ± 0.097	0.917 ± 0.086
PROTT5-AA	0.921 ± 0.073	0.910 ± 0.092	0.938 ± 0.051
PROSTT5-AA	0.948 ± 0.060	0.942 ± 0.069	0.954 ± 0.052
PROSTT5-3Di	0.949 ± 0.055	0.942 ± 0.062	0.959 ± 0.044
3Di-COMP	0.934 ± 0.098	0.915 ± 0.140	0.954 ± 0.065
3Di-KMER2	0.967 ± 0.046	0.961 ± 0.054	0.968 ± 0.044
3Di-KMER3	0.899 ± 0.078	0.891 ± 0.088	0.912 ± 0.073

## Appendix D

**Table A3:** Overview of machine learning features, along with their input data, the type of information they contain, and feature vector dimensions. Amino acid sequences were retrieved from Uniprot and used to calculate k-mer frequencies as well as PSSMs. The PSSM feature contains evolutionary exchange likelihoods for all 20 amino acid types found in protein sequences, calculated individually for each protein. The Multi-PSSM feature is then created by concatenating the four PSSM features into one vector. 3Di sequences were calculated with FoldSeek on predicted protein structures retrieved from AlphaFoldDB. In the COMB features, k-mers derived from the amino acid sequence and predicted 3D structure are combined. The protein language model (pLM) embeddings were mean-pooled along the sequence in order to turn them into per-protein embeddings. The non-informative DUMMY feature contains random values drawn from a uniform random distribution, and is used to estimate the additional information that is provided by each of the real features calculated from protein data.

Feature Name	Data Source	Type	Dimension
DUMMY	numpy.random	Unif. random values	1024
AAC	Amino acid sequence	1-mer frequencies	20
PAAC	Amino acid sequence	2-mer frequencies	400
AA-KMER3	Amino acid sequence	3-mer frequencies	8000
PSSM-50-1	PSIBLAST (Uniref50, 1 iteration)	Evol. information	400
PSSM-50-3	PSIBLAST (Uniref50, 3 iterations)	Evol. information	400
PSSM-90-1	PSIBLAST (Uniref90, 1 iteration)	Evol. information	400
PSSM-90-3	PSIBLAST (Uniref90, 3 iterations)	Evol. information	400
MULTI-PSSM	PSIBLAST (Four PSSMs)	Evol. information	1600
META	AAC + PAAC + MULTI-PSSM	Combined	2020
META-STD	META, standardized samples	Fingerprint	2020
3Di-COMP	3Di sequence	1-mer frequencies	20
3Di-KMER2	3Di sequence	2-mer frequencies	400
3Di-KMER3	3Di sequence	3-mer frequencies	8000
COMB-KMER1	AAC + 3Di-COMP	1-mer frequencies	40
COMB-KMER2	PAAC + 3Di-KMER2	2-mer frequencies	800
COMB-KMER3	AA-KMER3 + 3Di-KMER3	3-mer frequencies	1600
PROTT5-AA	Amino acid sequence	pLM embedding	1024
PROSTT5-AA	Amino acid sequence	pLM embedding	1024
PROSTT5-3Di	3Di sequence	pLM embedding	1024
